# Nine fatal cases of dengue: a case series from an intensive care unit in Sri Lanka

**DOI:** 10.1186/s41182-024-00661-w

**Published:** 2024-11-29

**Authors:** Pramith Ruwanpathirana, Harindri Athukorala, Thamalee Palliyaguru, Praveen Weeratunga, Dilshan Priyankara

**Affiliations:** 1 Professorial Unit in Medicine, National Hospital of Sri Lanka, Colombo, 00800 Sri Lanka; 2 Department of Clinical Medicine, Faculty of Medicine, University of Colombo, Colombo, Sri Lanka; 3 Medical Intensive Care Unit, National Hospital of Sri Lanka, Colombo, 00800 Sri Lanka

**Keywords:** Acute liver failure, Dengue, Death, Shock, Case series, Critical care, Infection

## Abstract

**Background:**

The case fatality rate of untreated dengue is 20%; it can be reduced to less than 1% with optimal management. The leading causes of death in dengue patients are shock, bleeding, and acute liver injury. We describe the clinical features of patients who died of dengue and discuss the therapeutic challenges and pitfalls of complicated dengue.

**Methods:**

This retrospective study was done in the intensive care unit (MICU) of the National Hospital of Sri Lanka over 30 months between 2021 and 2023. All patients who died of serologically confirmed dengue were incorporated.

**Results:**

Of the 1722 ICU admissions, 44 (2.6%) patients were treated for dengue—of them, 11 (25.0%) died. Two patients were excluded as their deaths were not directly linked to dengue. Six were females. The average age was 40.2 years. The leading causes of death included shock (*n* = 5), acute liver failure (*n* = 6), intracranial bleeding (*n* = 2), and pulmonary embolism (*n* = 1). Patient 1 had concomitant leakage and bleeding, which did not respond to fluids or blood products. He developed fluid overload and acute liver failure (ALF) and died of multiorgan dysfunction. Patients 2–5 were in shock for a prolonged period due to leakage ± bleeding. Patients 2–5 developed ALF and lactic acidosis followed by multiorgan dysfunction. Patient 8 developed acute hepatitis and ALF without preceding shock. The patient was treated with immunosuppressants for myasthenia gravis. Patients 6 and 7 experienced intracranial bleeding. Patient 9 died of pulmonary embolism after prolonged ventilation for dengue encephalitis.

**Conclusions:**

Prolonged shock, fluid overload and acute liver failure were common causes of dengue related deaths, in our study. Fluid overload occurred when vigorous crystalloid resuscitation was continued in patients who were poorly responding. A prompt switch to colloids or blood could have prevented overload. Patients who were in shock for a prolonged period become unresponsive to fluid resuscitation. How to manage dengue in patients who take anti-inflammatory drugs, immunomodulators, or antiplatelets is not known. Balancing the bleeding risk of dengue in patients predisposed to bleeding or thrombosis is a challenge.

## Introduction

Dengue, a major global public health challenge in the tropics and subtropics [1], has a mortality rate of 20% when left untreated and can be reduced to <1% with optimal management [2]. It mainly affects children and young adults [3], with an estimated annual economic burden of US $950 million in Southeast East Asia [4] and US $2·1 billion in the Americas [5]. Dengue is caused by four viral serotypes, DENV 1–DENV 4 [6], and is transmitted by the vector mosquitoes *Aedes aegypti* and *Aedes albopictus*.

**Fig. 1:**
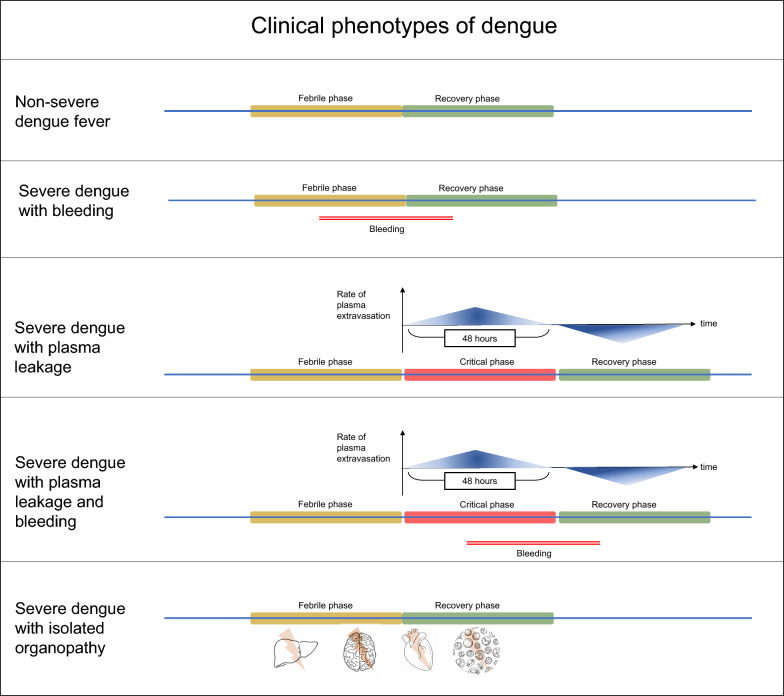
Clinical phenotypes of dengue. Non-severe dengue has two phases and does not lead to complications. In severe dengue, a critical phase includes the febrile and recovery phases. Plasma leakage occurs in the critical phase, which lasts for 48 h. The rate of leaking peaks approximately 24 h after the onset of the critical phase. Extravasated fluid is reabsorbed after the critical phase. Bleeding can occur in the presence or absence of plasma leakage. Dengue can cause isolated organopathy affecting the heart, brain, muscles, liver, and haematological system. Organopathy can coexist with leakage/bleeding

The clinical manifestations of dengue infection are heterogeneous. It ranges from an asymptomatic state, self-limiting acute febrile illness (non-severe dengue), an isolated organopathy (dengue encephalitis), to a severe dengue characterised by plasma leakage with or without bleeding (Fig. 1) [7]. The natural history of dengue fever is described in phases [7]. Patients with a self-limiting fever have 2 phases (febrile and recovery), and patients with severe dengue have three phases (febrile, critical, and recovery). The febrile phase is characterised by high-grade fever, headache, vomiting, and body aches. It lasts for 3–7 days. At the end of the febrile phase, patients with severe illness enter the critical phase. The critical phase is characterised by plasma leakage from the intravascular compartment to the extravascular compartment. The degree of plasma leakage varies from patient to patient and, if severe, amounts to hemodynamic compromise and shock. The critical phase classically lasts 48 h, and plasma leakage peaks at 24 h. An increase in packed red cell volume (PCV) parallels plasma leakage [8], indicating haemoconcentration, whereas bleeding reduces the PCV [7]. Extravasated fluid is reabsorbed into vessels at the end of the critical phase. Managing severe dengue involves maintaining adequate intravascular volume during the critical phase [9]. However, overzealous fluid therapy in the critical phase can lead to intravascular fluid overload when the extravasated fluid is reabsorbed. Bleeding can occur in the critical phase along with plasma leakage or rarely in the absence of plasma leakage.

The World Health Organization (WHO) classifies dengue as ‘dengue with or without warning signs’ and ‘severe dengue’ (Table 1) [7]. This classification was derived to aid clinicians in deciding where and how intensively the patient should be observed and treated, especially in outbreaks [7]. We have described the cases using the pathophysiological phases described above to appreciate the natural history of dengue.

**Table 1:** WHO disease classification scheme[7]

Dengue without warning signs	(1) Fever and two of the following: • Nausea, vomiting • Rash • Aches and pains • Leukopenia • Positive tourniquet test • Laboratory confirmed dengue (2) With ability: • To tolerate adequate volumes of oral fluid replacement • To pass urine at least once every 6 h
Dengue with warning signs	Patients with any of the following features: (1) At least one of the following warning signs: - Abdominal pain or tenderness - Persistent vomiting - Clinical fluid accumulation - Mucosal bleed (gingival bleeding, epistaxis, conjunctival bleeding, hematemesis, melena, fresh blood per rectum, haematuria, or vaginal bleeding) - Lethargy/restlessness - Liver enlargement > 2 cm - Increased haematocrit with a concurrent decrease in platelet count (≤100,000 platelets/mm^3^) OR (2) At least one comorbid condition, such as: - Pregnancy - Infancy - Old age - Diabetes mellitus - Renal failure OR (3) Social circumstances such as - Living alone - Living far from the hospital
Severe dengue	Patients with any of the following features: (1) Severe plasma leakage leading to: • Shock • Fluid accumulation leading to respiratory distress (2) Severe bleeding as evaluated by clinician (3) Severe organ involvement: • Liver: AST or ALT ≥ 1000 IU/L • Central nervous system: impaired consciousness • Heart and other organs

*AST* aspartate transaminase, *ALT* alanine transaminase

Progressive leukopenia and thrombocytopenia occur in the febrile phase [10]. This happens across the spectrum of illness (both severe dengue and self-limiting illness). In patients who progress to the critical phase, the onset of plasma leakage occurs approximately when the platelet level crosses 100 × 10^3^/µl [11]. The leucocyte count starts to increase from the onset of the critical phase. A neutrophil leucocytosis is observed in patients who develop shock.

A systematic review and meta-analysis of 150 studies identified 25 risk factors for the development of severe disease [12]; female sex, preexisting comorbidities, a lower platelet count within the first 4 days, a higher AST, and ALT levels were some of the risk predictors.

We planned to audit the causes of death in patients with dengue, admitted to the medical intensive care unit of the National Hospital of Sri Lanka. We critically analysed the therapeutic challenges, gaps in knowledge and the errors we have made, that would have contributed to the fatality. We expect that the findings of this study will help clinicians facing similar situations in the future to make the correct therapeutic decisions. Furthermore, we hope that the knowledge gaps highlighted in this study will stimulate future researchers.

## Methods

We performed a retrospective analysis on patients with dengue fever admitted to the medical intensive care unit (MICU) of the National Hospital of Sri Lanka in the years 2021–2023. We excluded 6 months when the MICU was dedicated to COVID-19. Only patients with a confirmed diagnosis of dengue were included. Serological confirmation was done with either immunochromatography (rapid antigen kit) or a fourfold rise of titre in the dengue antibodies (IgG or IgM) over 2 weeks assessed using the standard enzyme-linked immunosorbent assay. Patients who had dengue but died of nonrelated illnesses were excluded.

All biochemical and haematological testing was done in the laboratory of the National Hospital of Sri Lanka. Bedside ultrasound was performed using standardised protocols for dengue [13]. This included views of the gall bladder (for wall oedema), hepatorenal space, splenorenal space, pouch of Douglas and bilateral pleural spaces. Ultrasonic evidence of plasma leakage was defined as free fluid >2 mm in depth in any of these spaces. All ultrasounds were performed by physicians with at least 2 years of experience in dengue ultrasonography.

We developed a data collection sheet that included demographic data, clinical, biochemical, and haematological parameters on admission to the hospital, clinical progression of the illness, and management both before and after admission to the ICU. The haemodynamic parameters, the amount and type of fluid administered, the response to fluid therapy and the use of iono-pressers in the clinical phase was documented in detail. We charted the sequential biochemical and laboratory parameters and correlated them with the clinical progression and therapy. Three investigators (PR, HA, and TP, who were specially trained in dengue and were involved in conceptualisation) individually collected the details from the patients’ clinical records. Two senior investigators (DP and PW) reviewed the areas of discrepancy, recollected the data from the clinical records and finalised the data collection sheet. We did not calculate the kappa statistic for inter-rater reliability. As dengue management is protocolised by the Ministry of Health, Government of Sri Lanka (General Circular 01–49/2012), there was uniformity in the clinical record keeping [14]. Hence, there were no missing data. Because of the heterogeneity of the causes of death and the limited number of patients, we did not perform a statistical analysis for associations with mortality. We provide a detailed description of the clinical progression of each patient and emphasise the potential pitfalls and therapeutic challenges for the reader. We have discussed the confounding factors for the causality of death on a case-to-case basis. Ethical approval for this retrospective analysis was obtained from the ethics review committee of the Faculty of Medicine, University of Colombo.

We defined acute liver failure (ALF) as ‘evidence of coagulation abnormality, usually an International Normalized Ratio (INR) > 1.5, and any degree of mental alteration (encephalopathy) in a patient without preexisting cirrhosis and with an illness of <26 week duration’ as per the American Association for the Study of Liver Disease [15]. Although all causes of acute liver failure (such as hepatitis viral infection, Wilson’s disease, etc.) were not excluded in all patients, we assumed new-onset ALF that paralleled the clinical illness to be due to dengue until proven otherwise. Bleeding was diagnosed either when the source was obvious or when the source was occult, but there were haematologically suggestive features such as dropping PCV with worsening haemodynamics.

## Results

**Fig. 2:**
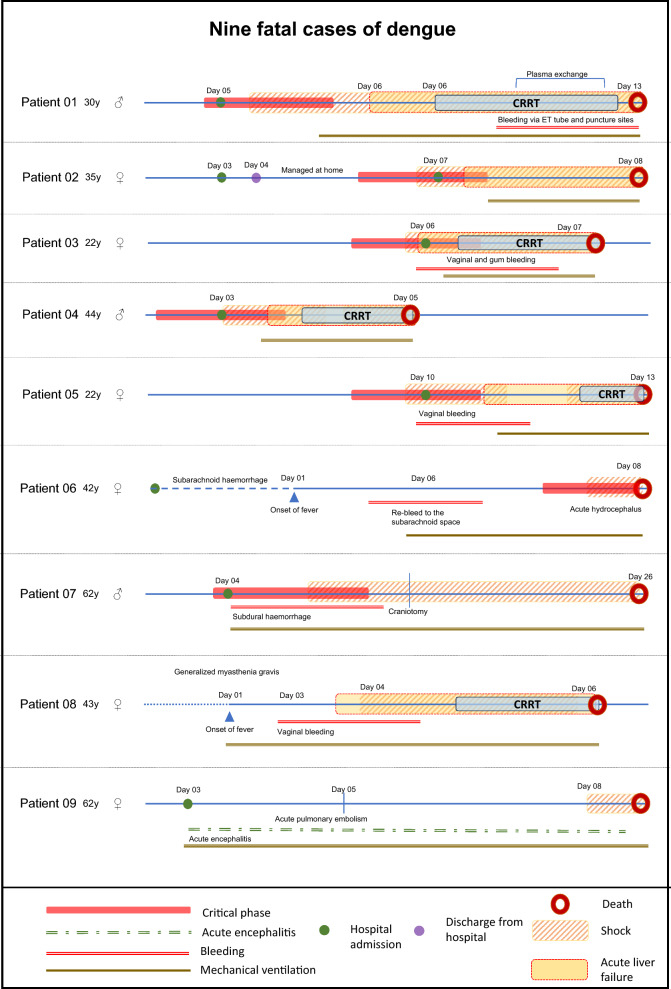
Timelines of events of the nine patients

Of the 1722 ICU admissions over 30 months, excluding the period dedicated to COVID-19, 44 (2.6%) patients were treated for dengue. Of them, 11 (25.0%) died. Nine patients were selected for this review (Fig. 2). Of the two patients not included in this review, one died of pneumothorax after mechanical ventilation for severe pneumonia; he developed dengue with leaking but no end-organ injury. The other patient was managed in another hospital and died due to a sudden cardiac arrest. Because we could not establish whether the patient had dengue myocarditis or whether the cardiac arrest was related to another cause, we excluded him from the analysis.

Six of the nine patients were females, and the average age of the sample was 40.2 years. Seven patients were transferred from regional hospitals, while the rest were admitted directly to the National Hospital of Sri Lanka.

## Case presentations

### Patient 1: refractory shock, fluid overload, and acute liver failure

A 30-year-old previously healthy man presented with fever, headache, diarrhoea, and vomiting for 5 days. A medical practitioner had not reviewed him before the presentation but had taken over-the-counter mefenamic acid and diclofenac sodium. On admission, his haemodynamic parameters were normal. The white cell count was 11.49 × 10^3^/µl (neutrophil—6.2 × 10^3^/µl, lymphocyte—5.2 × 10^3^/µl), and the platelet count was 12 × 10^3^/µl. The packed red cell volume (PCV) was 49%. The bedside ultrasound scan was negative for fluid leakage. He was treated with 100 ml/h IV normal saline for maintenance and additional fluid per loose stool or vomitus. The dengue NS1 antigen was positive.

Four hours later, the patient became anuric, and a pulse rate of 120 beats per minute was noted. His blood pressure decreased to 100/80 mmHg, with a 30 mmHg postural drop in the systolic blood pressure. The PCV decreased to 41%. He was treated with a 500 ml bolus of 0.9% saline followed by a tapering regimen of crystalloids. A repeat ultrasound scan revealed bilateral pleural effusions and free fluid in the abdomen. Subsequently, the blood pressure decreased to 70/50 mmHg, and the heart rate rose to 155 beats/minute despite fluid resuscitation. Over 1 h, five hundred millilitres of 40% dextran were given to replenish the intravascular volume. The PCV decreased to 25%, after which the packed red blood cells were transfused. Despite blood transfusion, the PCV continued to decline. We diagnosed concomitant plasma leakage and concealed bleeding.

**Fig. 3:**
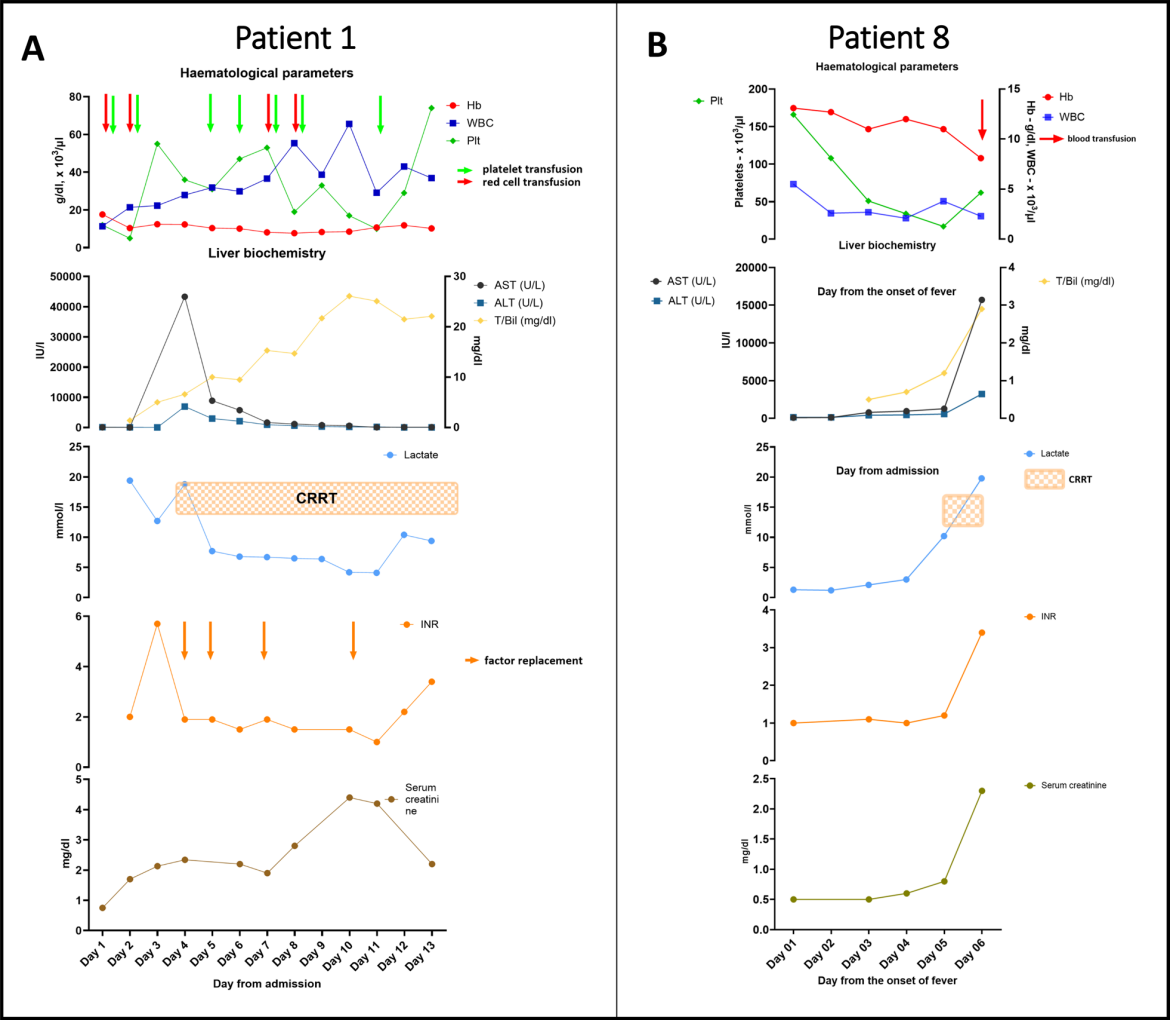
Progression of laboratory parameters in patients 1 and 8

He became progressively drowsy and was intubated at the 13th hour after admission. Over the next 20 h, he was transfused with five units of packed red cells, 11 units of platelets, 12 units of fresh frozen plasma, and 800 ml of cryoprecipitate. The total volume of fluids infused in the first 20 h after admission was 6910 ml. At this point, the patient was still in shock and had fluid overload. Fluid infusions were stopped, and therapy with vasopressors was initiated. No overt bleeding was identified. The patient had worsening lactic acidosis (pH 7.0, serum lactate = 19.4 mmol/l, HCO_3_^−^ = 13.1 mmol/l). Continuous renal replacement therapy was initiated. A negative fluid balance was maintained with the CRRT, as he was oliguric. The progression of haematological and biochemical parameters is given in Fig. 3A.

The jugular venous pressure was elevated 7 cm from the sternal notch. The patient’s blood pressure was 80/50 mmHg, and his heart rate was 160 beats/minute. There were bilateral moderate to large pleural effusions and ascites. The lung fields had multiple B-lines bilaterally. The inferior vena cava was distended with a left ventricular ejection fraction of 30% in the bedside echocardiography. His blood sugar and serum calcium levels were within the normal range.

Peritoneal fluid was aspirated with a rigid peritoneal dialysis catheter, where 500 millilitres of blood-stained fluid was drained. The patient started bleeding from the endotracheal tube and puncture sites. Rotational thromboelastometry revealed a marked prolongation in intrinsic thromboelastometry (INTEM), extrinsic thromboelastometry (EXTEM), and FIBTEM (EXTEM with platelet inhibitors). His serum fibrinogen level was 0.7 g/l (1.8–3.5). There was no evidence of microangiopathic haemolytic anaemia in the blood. He progressed to acute hepatic failure. Therapeutic plasma exchange was initiated as a treatment for acute liver failure, but the patient died on the 13th day of illness.

### Patients 2–5: prolonged shock

#### Patient 2

A 35-year-old female presented with fever, arthralgia, and myalgia for 7 days. On admission, she was in shock with a blood pressure of 90/60 mmHg and a pulse rate of 120/min, with low volume. There was ultrasonic evidence of plasma leakage; to the hepatorenal pouch and pouch of Douglas. The platelet count and total leucocyte count on admission were 61 × 10^3^/µl and 3.5 × 10^3^/µl, respectively. Despite intensive fluid resuscitation, her haemodynamic parameters did not improve, and she progressed to acute liver failure.

Her blood pressure was unrecordable on admission to the ICU. She was treated with IV crystalloids, blood products, IV albumin, IV sodium bicarbonate, and three iono-pressors (noradrenaline, vasopressin, and dobutamine). N-acetyl cysteine was administered for acute liver failure. Despite this, she died on the eighth day after the onset of the fever.

In retrospect, she presented to the local hospital on the third day of fever but was discharged because her platelet and leucocyte counts were 316 × 10^3^/µl and 5 × 10^3^/µl, respectively. Her NS1 antigen was positive. She had no high-risk features (bleeding manifestations, abdominal pain, vomiting, or diarrhoea). She was discharged with the advice for outpatient monitoring. Due to multiple socioeconomic pressures, she did not comply with the advice and presented with symptoms of shock.

#### Patient 3

A 22-year-old previously healthy female presented with a fever for 6 days and bleeding from the vagina and oral mucosa. Dengue NS-1 antigen was positive. On admission, she had evidence of plasma leakage and haemodynamic compromise. The pulse was thready at a rate of 142 beats/min, and the capillary refill time was prolonged by 3 s. Her blood pressure was 120/100 mmHg. There was a right-sided moderate pleural effusion.

The total leucocyte and platelet counts were 7.5 × 10^3^/µl (neutrophil—5.3 × 10^3^/µl, lymphocyte—1.2 × 10^3^/µl) and 11 × 10^3^/µl, respectively, on admission. There was evidence of haemoconcentration with a packed red cell volume of 51% and a haemoglobin level of 17 g/dl. On admission, the aspartate transaminase (AST) and alanine transaminase (ALT) levels were 2212 IU/l and 719 IU/l, respectively. Despite fluid resuscitation with crystalloids, colloids, tranexamic acid, and blood products, the patient progressed to acute liver failure with refractory acidosis. The patient was intubated and ventilated. Continuous renal replacement therapy was initiated. She died on the second day after admission.

#### Patient 4

A 44-year-old male patient with diabetes and hypertension presented with fever for 3 days with arthralgia, myalgia, and lassitude for 1 day. On admission, he was drowsy, with cold peripheries and an unrecordable blood pressure. The packed red cell volume was 65%. Bedside ultrasound revealed free fluid in the hepatorenal pouch and bilateral moderate pleural effusions. The serum lactate concentration was 13.8 mmol/l, and the pH was 7.0 (HCO_3_^−^ = 4.9 mmol/l). His blood sugar was 200 mg/dl.

Vigorous resuscitation with IV crystalloids was initiated. After 500 ml of 0.9% NaCl, his blood pressure improved to 160/110 mmHg. The IV fluid was gradually tapered off, and IV isotonic NaHCO3 was used to treat the acidosis. Despite resuscitation, he continued to be anuric. Continuous renal replacement therapy was initiated due to worsening lactic acidosis. He progressed to acute liver failure, with AST increasing  from 9494 to 41,057 IU/l and ALT increasing from 3232 to 10,424 IU/l, over 24 h. His INR was 6.5, and the creatinine level was 2.7 mg/dl. The total leucocyte and platelet counts were 15.73 × 10^3^/µl (6.2 × 10^3^/µl neutrophils and 4.3 × 10^3^/µl lymphocytes) and 15 × 10^3^/µl, respectively. He was intubated due to a deteriorating level of consciousness. He succumbed to multiorgan failure on the fifth day of illness.

#### Patient 5

A 24-year-old female presented with fever, arthralgia, and myalgia for 10 days. Dengue NS1 antigen was positive on the second day of illness, and she was followed up as an outpatient. She got admitted on the tenth day with headache, nausea, vomiting, intermenstrual vaginal bleeding and reduced urine output. On examination, she was ill, with a pulse rate of 112 beats/min and a blood pressure of 70/50 mmHg. Bedside ultrasound revealed free fluid in the hepatorenal pouch and the pouch of Douglas. She was treated with IV crystalloids, which improved her blood pressure. However, she progressed to acute liver failure and lactic acidosis with secondary haemodynamic compromise, to which she succumbed. The maximum AST and ALT were 28,384 IU/l and 4759 IU/l respectively, and the INR was 6.1.

### Patient 6: subarachnoid haemorrhage

**Fig. 4:**
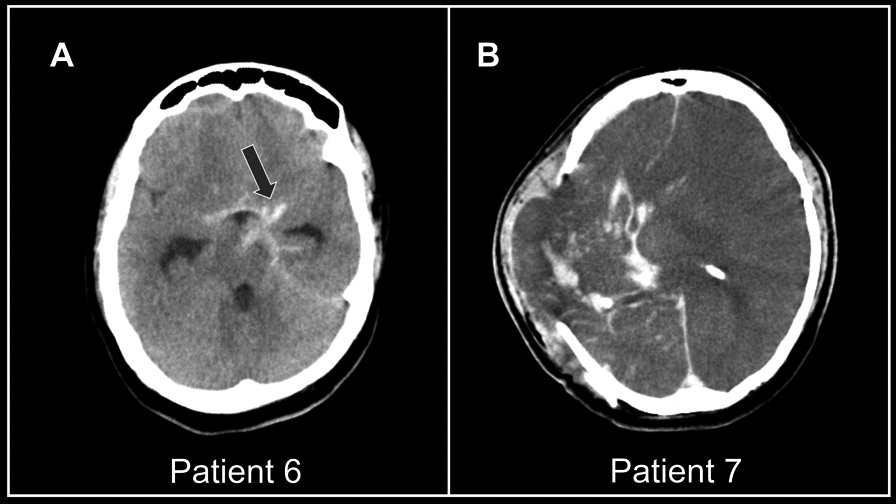
Brain imaging of patients 6 and 7. **A** Non-contrast CT image of the brain, as described for Patient 6, demonstrating subarachnoid haemorrhage (*arrow*) and acute hydrocephalus. **B** The postoperative non-contrast CT described in Patient 7 revealed right craniectomy, gross cerebral oedema, and right hemispherical herniation with intraparenchymal bleeding

A 42-year-old female presented with acute onset occipital headache and transient loss of consciousness followed by generalised tonic–clonic seizures. She was diagnosed with subarachnoid haemorrhage (SAH) secondary to a saccular aneurysm in the left internal carotid artery. Standard treatment with oral nimodipine, phenytoin, and blood pressure control was initiated. While awaiting embolisation, she developed a high-grade fever. Her dengue NS-1 antigen was positive on the second day after the onset of fever. There was progressive thrombocytopenia despite platelet transfusion—on the sixth day, the patient developed rebleeding into the subarachnoid space, resulting in a progressive reduction in consciousness (Fig. 4A). She was intubated for airway protection. She developed dengue shock on the seventh day, which was resistant to fluid and inotrope therapy. Surgery was postponed due to ongoing infection and haemodynamic instability. She died on the eighth day after the onset of fever from acute hydrocephalus.

### Patient 7: Subdural haemorrhage

A 62-year-old male presented with acute onset fever with arthralgia and myalgia for 4 days, followed by acute loss of consciousness. His NS1 antigen was positive on the second day of illness and was managed at home. On the fourth day, he was found unconscious. His GCS score was three on admission, with a peripheral oxygen saturation of 81%. He was intubated immediately. His blood sugar was 145 mg/dl, blood pressure was 219/97 mmHg, and pulse rate was 54 beats/minute. The pupils were nonreactive (5 mm bilaterally). There was no papilledema. The reflexes were globally diminished with an extensor plantar response.

He had a history of diabetes, hypertension, and ischaemic heart disease, treated with aspirin and clopidogrel, among other drugs. The patient continued taking antiplatelets even after the onset of the fever.

His total leucocyte, platelet, and haemoglobin levels were 7.8 × 10^3^/µl, 64 × 10^3^/µl, and 15.2 g/dl, respectively. Bedside ultrasound revealed plasma leakage into the hepatorenal pouch. There was a large right-sided frontal–parietal–temporal subdural haemorrhage (SDH) with midline shift and cerebral oedema—in the brain scan. Craniotomy and SDH evacuation were performed after platelet transfusions (postoperative CT—Fig. 4B). However, the level of consciousness did not improve. His blood pressure progressively declined despite treatment with IV crystalloids and iono-pressors, and he succumbed to refractory shock on the eighth day of illness.

### Patient 8: myasthenia gravis and dengue

A 43-year-old female with generalised myasthenia gravis (MG) treated with pyridostigmine, prednisolone, and azathioprine developed fever with arthralgia and myalgia while being treated for a myasthenic crisis with intravenous immunoglobulins (IVIGs). She was found to have increased transaminase levels upon admission before the onset of fever, which was attributed to azathioprine-induced acute liver injury (AST = 24 IU/l, ALT = 84 IU/l). Along with the development of fever, there was a progressive deterioration in respiratory muscle power, and the patient was intubated.

On the second day after the onset of the fever, she was diagnosed with dengue with a positive NS1 antigen. Treatment for MG with IVIG, pyridostigmine, and prednisolone continued. She developed per-vaginal bleeding on the third day of fever, but her haemodynamic parameters were normal. The progression of laboratory parameters is given in Fig. 3B. She did not have radiological evidence of plasma leakage.

On the fourth day after the onset of the fever, her blood pressure started to decrease, with a poor response to fluid therapy requiring vasopressor support with IV noradrenaline. The bedside scan was still negative for free fluid in the abdomen and pleura. Her AST and ALT increased to 15,709 and 3229 IU/l, respectively, and the serum creatinine increased to 2.3 mg/dl from 0.6 mg/dl. There was severe metabolic acidosis with a pH of 6.5 (HCO_3_^−^ = 7.8 mmol/l), which was refractory to treatment with IV NaHCO_3_. Despite initiating renal replacement therapy, she died on the sixth day secondary to multiorgan failure.

### Patient 9: dengue encephalitis and pulmonary embolism

A 62-year-old female presented with a fever for 3 days and altered behaviour for 2 days. Her dengue antigen test was positive. Due to the progressive deterioration of consciousness, she was intubated. Her EEG showed diffuse slowing suggestive of encephalopathy. MRI of the brain was normal. Cerebro-spinal fluid (CSF) analysis revealed a lymphocytic reaction and high protein (115 mg/dl) without a decrease in sugar (CSF sugar 209 mg/dl, RBS 250 mg/dl). Dengue antibodies were present in the CSF. The CSF culture was sterile, and antigen testing was negative for meningococcus, pneumococcus, and *Haemophilus*. Polymerase chain reaction for *Mycobacterium tuberculosis* DNA was negative.

Her haemodynamic parameters were stable throughout, and she did not develop plasma leakage. Her consciousness level was slow to recover. She died on the seventh day of illness secondary to a pulmonary embolism confirmed by a CT pulmonary angiogram. She was not on pharmacological prophylaxis for venous thromboembolism due to the increased risk of bleeding from dengue fever.

## Discussion

Hepatic failure, dengue shock syndrome, and encephalopathy are the leading causes of death in dengue worldwide [16]. We describe nine fatal cases of dengue. Six patients developed acute liver failure: 5 due to dengue shock syndrome and one due to dengue hepatitis. Two patients died of complicated intracranial haemorrhages, and one died of pulmonary embolism after prolonged immobilisation for dengue encephalitis.

### Difficult to treat shock and ischaemic liver failure

Patient $1$ portrays the natural history of a patient with difficult-to-treat shock. He presented with normal haemodynamic parameters but progressed to shock within 4 h. Although he was aggressively resuscitated with crystalloids, his haemodynamic did not improve. This fluid non-responsiveness indicates severe leakage where the infused fluid leaks from the vascular compartment. Although we expect a decrease in PCV with fluid resuscitation, it should parallel clinical and hemodynamic improvements [7]. In Patient 1, the PCV decreased while his haemodynamic deteriorated. This indicates bleeding. Concealed bleeding is a known manifestation of dengue [9].

Extensive leakage promotes aggressive fluid therapy, which can result in fluid overload [17]. Fluid overload can coexist with shock: excess fluid fills the extravascular compartments [18]. The risk of overload increases when more than 4600 ml of fluid is given during the 48 h of the critical phase in patients with more than 50 kg [11]. The features of fluid overload are peri-orbital oedema, abdominal distention, tachypnoea, respiratory distress with lung signs of pulmonary oedema, and tachycardia [11].

The admission ultrasound scan of patient 1 did not reveal evidence of plasma leakage. However, the patient was likely already in the critical phase upon admission. A very high PCV (49%) is a tell-tale sign of plasma leakage and haemoconcentration [7]. Furthermore, the patient progressed to shock within 4 h. Shock is expected to occur when the rate of plasma extravasation reaches a maximum, i.e., 24 h from the onset of the critical phase [7, 11]. The absence of ultrasonic evidence of fluid in the extravascular spaces does not exclude vascular leakage, especially in patients with dehydration (Ex—vomiting, diarrhoea) [19, 20]—where the intra-vascular volume is depleted. Therefore, the net amount of plasma leaked is less despite the increasing vascular porosity during the critical phase due to low hydrostatic pressures. Hence, plasma leakage might not be detected ultrasonically in dehydrated patients. A disproportionately high PCV to the degree of dehydration should alert the physician of vascular leakage. The appearance of pleural effusions and ascites immediately after fluid resuscitation indicates established vascular porosity. Therefore, it is advisable to repeat bedside leaking scans in patients who present with dehydration/shock after fluid resuscitation if the initial leaking scans are negative.

Both shock (hypovolemia and haemorrhage) and fluid overload can lead to acute liver injury and progressive liver failure [21].

Patients 2–5 presented with prolonged shock. All patients developed acute liver failure and died of complications. We have observed that patients who present with protracted shock progress to liver injury despite fluid resuscitation (unpublished data). This finding suggests the presence of a therapeutic window in the early hours of the critical phase to treat shock and prevent end-organ damage.

Liver failure is the terminal pathway in most patients with critical dengue. The transaminase concentration increases to above 1000 IU/l over 3–6 days [21]. The AST is higher than ALT [22, 23]. In addition to being released from hepatocytes, AST is released from skeletal and cardiac muscles and erythrocytes. This might explain the higher AST than ALT [24] in dengue. The transaminase level decreases over the next few days while the serum bilirubin level and INR increase [21]. Lactic acidosis is an early marker of liver injury [25]. Hyperlactatemia results from anaerobic metabolism due to tissue hyperperfusion and reduced clearance due to hepatic dysfunction [25]. Fulminant hepatic failure and acidosis are soon followed by cardiac dysfunction, coagulopathy, encephalopathy, acute kidney injury, shock, and death.

The ideal management of acute liver failure in dengue is not known. Liver transplantation was performed for acute liver failure in dengue patients [26]. However, routine liver transplantation is challenged by haemodynamic instability, bleeding risk, and hyperacute progression of the illness. Lactic acidosis is often resistant to IV HCO_3_^−^ therapy. Fluid management is confounded by the mandatory volume required for IV HCO_3_^−^ therapy. Therefore, we resort to continuous renal replacement therapy (CRRT) for patients with severe acidosis and haemodynamic instability. The CRRT provides additional control over fluid balance. We treat patients with IV N-acetyl cysteine (NAC) based on the evidence of paracetamol poisoning. A Sri Lankan study with 30 patients demonstrated a clinical and biochemical improvement with NAC [27].

Wijewickrama et al*.* demonstrated that NSAIDs taken during illness increase the risk of bleeding and hepatic enzyme derangement [28]. Patient 1 had taken NSAIDs during the illness, which might have amplified the disease.

### Dengue hepatitis and dengue in immunosuppressed patients

Patient 8 progressed to acute liver failure without haemodynamic compromise. The pathophysiology of liver failure in dengue patients is not entirely understood. When it occurs following shock, it is likely due to hepatic ischemia. However, dengue hepatitis and hepatic failure are described in the absence of shock. Dengue is a hepatotropic virus. Dengue viral antigens and RNA have been detected in hepatocytes and Kupffer cells [29–31]. Mild to moderate transaminitis is common in dengue fever [24]. Patient 8 probably had dengue hepatitis due to direct viral invasion of the liver cells. We believe the immunodeficient status predisposed the patient to hepatitis, and the background liver injury promoted progression to fulminant liver failure. Prolonged viremia and viriuria have been noted in immune-suppressed patients with dengue [32].

The pathophysiology of vascular leakage and organ injury in dengue is not clear. However, it is postulated to be immune-driven [21, 33]. TNF-$\alpha$, IL-2, IL-6, IL-8, IL-12, and IFN-$\gamma$ are significantly elevated in dengue patients with leakage compared with those with uncomplicated dengue fever [34]. Patient 8 was treated with immunosuppressants and immunomodulators. The interaction between immunosuppressants and dengue is not clear. Numerous authors have discussed the effect of corticosteroids [35] and IVIG [36] in managing dengue.

### Intracranial bleeding in dengue patients

Patients 6 and 7 had fatal intracranial bleeding. In a retrospective study of 182 adult dengue patients who underwent brain computed tomography, 13 (7.14%) had intracranial haemorrhages [37]. A case series from India described three patients with basal ganglia haemorrhages and two patients with subdural haemorrhages [38].

Dengue poses several challenges in managing intracranial bleeding. An increased risk of intra-post-operative bleeding confounds surgical intervention. There is no consensus on managing vasculopathy, coagulopathy, or thrombocytopenia/thrombasthenia peri-operatively. Intracranial bleeding demands meticulous blood pressure control. Antihypertensives increase the risk of dengue shock by counteracting hemodynamic compensatory mechanisms. In a case series of 8 dengue patients with intracranial haemorrhages, five patients were successfully managed surgically [39]. The authors have transfused platelets peri-operatively. Patients with brain parenchymal bleeding were not operated on and had a fatal outcome.

Patient 7 continued to take antiplatelets after the onset of illness. The optimal management strategies for treating/preventing bleeding in such patients are unknown. Some unanswered questions in dengue are whether there is an indication for prophylactic platelet transfusion, when the antiplatelets should be withheld, and when it is safe to restart them.

### Thrombo-prophylaxis in dengue patients

A systematic review performed in 2022 described five patients with dengue complicated with thrombosis—four with deep vein thrombosis in the lower limbs and one with portal vein thrombosis [40]. Venous thromboembolism prophylaxis is a therapeutic challenge in managing critically ill dengue patients. Pharmaco-prophylaxis increases the risk of bleeding, whereas non-pharmacological methods might not be effective in the case of prolonged immobilisation. Case 9 describes thrombosis in the sequelae of complicated dengue. The point at which the risk of bleeding in dengue patients diminishes and when to start prophylactic anticoagulation therapy is unknown.

Dengue virus is associated with numerous neurological complications [41, 42], such as encephalitis, transverse myelitis, acute disseminated encephalomyelitis [43], Guillain‒Barré syndrome [44], and myositis. The presence of dengue virions and anti-dengue IgM antibodies in the CSF suggests neuro-tropism of the dengue virus [41]. Brain MRI is normal in 23.8% of patients with dengue encephalitis [45]. Dengue encephalitis is a self-limiting illness with good clinical recovery [46].

We have summarised a critical analysis of the above patients and highlighted the medical errors, learning points and gaps in knowledge in Table 2.

**Table 2:** Medical errors, learning points and gaps in knowledge identified in the critical analysis of the nine patients

Patient number	Medical errors identified	Learning points from identified errors	Gaps in knowledge
Patient 01	*Excluding plasma leakage with a single bedside ultrasound scan (USS)* The patient was managed as in the febrile phase based on a single ultrasound scan, despite having a haemodynamic compromise	The absence of ultrasonic evidence of fluid leaking does not equate to the absence of fluid leaking. If the clinical suspicion is high, USS must be repeated after fluid resuscitation	The impact of non-steroidal anti-inflammatory drugs on the natural history of dengue^a^
	*Sub-optimal initial fluid resuscitation* Despite evidence of intra-vascular volume depletion, the patient was given a fixed regimen of IV saline 100 ml/h	The fluid replacement regimen should be tailor-made to the patient’s haemodynamic status	What is the mechanism of refractory shock in prolonged dengue shock?
	*Delayed detection of bleeding* The patient’s packed cell volume (PCV) dropped to 41% from 49% while being haemodynamically unstable. This indicates bleeding. But, the bleeding was not detected till the PCV dropped to 25%	Bleeding should be suspected when the patient’s PCV drops (or does not increase) while the haemodynamic parameters are worsening Bleeding in dengue is not always obvious. Occult bleeding is common	
	*Wrong choice of replacement fluid* When the patient was not responding to crystalloid resuscitation, the replacement fluid should have been changed to a colloid. Blood should have been transfused early, given the occult bleeding	When there is a poor response to crystalloids, the replacement fluid should be changed to a colloid The preferred colloid in the presence of bleeding is blood The preferred fluid for a patient who has received a substantial amount of crystalloids (in the absence of bleeding) is 40% dextran	
	*Fluid overload* The main reason for fluid overload in this patient was the inappropriate choice of replacement fluid. Because the patient did not respond adequately to crystalloid therapy, more and more crystalloids were given, resulting in overload	Clinicians should be mindful of the fluid quota not to be exceeded within the critical phase^b^. If the patient is likely to require crystalloids exceeding the quota (due to poor response), colloids should be used instead	
	*Prolonged shock* Prolonged shock predisposes to refractory hypotension	Shock in dengue should be treated early. Once it reaches the refractory state, no amount of fluid will reverse the disease course	
Patient 2	*Unsafe discharge* Medically, the patient was fit to be discharged for outpatient monitoring. However, her socio-economic background was suggestive of poor compliance	When discharging a patient with dengue fever for outpatient monitoring, the clinician should be mindful of the socio-demographic determinants of compliance with medical advice When in doubt, it is best to admit the patient for monitoring	
Patient 3 & Patient 5	*Delayed presentation* The patient presented late after the organ injury had already been established. After a prolonged shock, the patient enters a phase refractory to treatment	Public education on early screening for dengue in febrile illnesses is important; especially in endemic areas All acute febrile illnesses should be evaluated for dengue within the first 2–3 days	
Patient 4			This patient progressed to acute liver failure despite fluid resuscitation and correction of hypotension We hypothesise that the presence of metabolic dysfunction associated steatotic liver disease (MASLD) augmented the dengue-related liver injury. Further research is needed
Patient 6			Management of dengue in a patient with a leaking cerebral aneurysm is challenging. The exact pathophysiological alteration of the coagulopathy in dengue is not known. The place for platelet transfusion needs to be studied
Patient 7			How can the bleeding risk be mitigated in patients treated with antiplatelets who develop dengue? How can a patient with dengue be prepared for emergency surgery?
Patient 8			What is the impact of immunomodulators on the disease course of dengue? How do we differentiate dengue hepatitis from other causes of liver injury? How does dengue hepatitis differ from the liver injury seen in dengue shock syndromes?
Patient 9			How do we balance the risk of thrombosis in a patient with dengue?

^a^Non-steroidal anti-inflammatory drugs (NSAIDs) are usually not prescribed in dengue as it is believed that they increase bleeding by interfering with the platelet activation. There is scarce knowledge about the exact mechanism. Whether NSAIDs interact with endothelial function and worsen capillary leaking is not known. Studies to identify if the above hypothesis is true are needed.

^b^The total amount of fluid not to be exceeded during the critical phase (48 h) is calculated based on the ideal body weight. Fluid quota = (M + 50 ml x body weight (kg)). M = the weight-based fluid requirement calculated as 100 ml/kg for the first 10 kg + 50 ml/kg for the next 10 kg + 20 ml/kg for balance weight. The fluid quota is a rough guide. The entire quota need not be infused during the critical phase. It guides switching to a colloid when the patient will likely exceed the fluid quota.

*MASLD* Metabolic dysfunction associated steatotic liver disease, *PCV* Packed cell volume, *USS* Ultrasound scan

We could not perform a statistical analysis to identify predictors and indicators of mortality as the study sample was small and the causes of death were heterogeneous. We recognise this as a major limitation of the study. The sample used in the study over-represents patients with medically challenging critically ill dengue patients because many such patients are transferred to the study centre from all over Western Sri Lanka. Furthermore, we could not exclude many confounding illnesses that could contribute to mortality, such as preexisting liver disease in patients who developed liver-related complications. We could not serotype the dengue virus.

### Summary

This case series of fatal dengue highlights the importance of early recognition and management of plasma leakage and occult bleeding. The progression to acute liver failure is often preceded by prolonged shock and fluid overload. Our observations suggest a potential therapeutic window in the early critical phase where aggressive management might prevent irreversible organ damage. The challenges in managing intracranial bleeding and the risk of thrombotic complications in prolonged illness were also notable. The effect of steroids and immunosuppressants on dengue is not known. It is prudent to be extra cautious in managing immunosuppressed patients with dengue. These findings underscore the need for further research on fluid management strategies, early markers of severe disease progression, and specific therapies for dengue-associated acute liver failure. Improved understanding and management of these severe complications may help reduce mortality in dengue patients.

## Data Availability

The clinical data used in the current study are available from the corresponding author upon reasonable request.

## Abbreviations

ALF: Acute liver failure
ALT: Alanine transaminase
AST: Aspartate transaminase
COVID-19: Coronavirus disease
CRRT: Continuous renal replacement therapy
CSF: Cerebrospinal fluid
CT: Computed tomography
EEG: Electroencephalogram
EXTEM: Extrinsic thromboelastometry
ICU: Intensive care unit
IFN: Interferon
IL: Interleukin
INR: International normalised ratio
INTEM: Intrinsic thromboelastometry
IV: Intravenous
IVIG: Intravenous immunoglobulin
MG: Myasthenia gravis
NAC: N-acetyl cysteine
NS-1: Non-structural protein
NSAIDs: Non-steroidal anti-inflammatory drugs
PCV: Packed red cell volume
SAH: Subarachnoid haemorrhage
SDH: Subdural haemorrhage
TNF: Tumor necrosis factor
